# Data on nearshore wave process and surficial beach deposits, central Tamil Nadu coast, India

**DOI:** 10.1016/j.dib.2017.05.052

**Published:** 2017-06-03

**Authors:** V. Joevivek, N. Chandrasekar

**Affiliations:** a Centre for GeoTechnology, Manonmaniam Sundaranar University, Abishekapatti, Tirunelveli 627012, Tamil Nadu, India; b Akshaya College of Engineering and Technology, Kinathukadavu, Coimbatore 642109, Tamil Nadu, India

**Keywords:** Beach, Wave, Nearshore, Grain size, Placer minerals, India

## Abstract

The chronicles of nearshore morphology and surficial beach deposits provide valuable information about the nature of the beach condition and the depositional environment. It imparts an understanding about the spatial and temporal relationship of nearshore waves and its influence over the distribution of beach sediments. This article contains data about wave and sediment dynamics of the ten sandy beaches along the central Tamil Nadu coast, India. This present dataset comprises nearshore wave parameters, breaker wave type, beach morphodynamic state, grain size distribution and weight percentage of heavy and light mineral distribution. The dataset will figure out the beach morphology and hydrodynamic condition with respect to the different monsoonal season. This will act as a field reference to realize the coastal dynamics in an open sea condition. The nearshore entities were obtained from the intensive field survey between January 2011 and December 2011, while characteristics of beach sediments are examined by the chemical process in the laboratory environment.

**Specifications Table**

**Table:** 

Subject area	Earth science
More specific subject area	Oceanography
Type of data	Table, figure
How data was acquired	Field survey, sample collection and laboratory analysis
Data format	Raw data collection and Analysis
Experimental factors	The raw sediment samples were dried overnight at 105 °C and treated by H_2_O_2_ and Dil. HCl for removal of organic and inorganic matters. Further, about 100 g of sediments were refined by coning and quartering method for the purpose of grain size analysis.
Experimental features	Computational process: breaker wave type, beach morphodynamic state were computed from nearshore data by using ONWET statistical tool.
	Mechanical process: The size of grain distribution is classified by sieving the sample using Ro-top sieve shaker.
	Chemical process: Heavy and light minerals were isolated by addition of Bromoform (CHBr_3_) heavy liquid.
Data source location	A 51 km coastal stretch between Thirukadaiyur and Velankanni, central Tamil Nadu coast, India
Data accessibility	Data is with this article

**Value of the data**

• This dataset will act as guide for researchers, scientists, engineers, and environmentalists to realize the beach and nearshore process in a year under different monsoonal conditions.
• The nearshore wave data reveals the dominating forces that act on the shoaling region and its impact on the beach morphology.
• The breaker wave type and beach morphodynamic state provide a significant way to look at the effectiveness of nearshore waves with respect to the monsoon seasons.
• The grain size data highlights the wave energy condition and longshore sediment drift displayed in the coast.
• The weight distribution of placer deposits provide an insight to deposition of economic placer minerals in accordance with the beach and wave condition.

## 1 Data, experimental design, materials and methods

### 1.1 Data

**Fig. 1:**
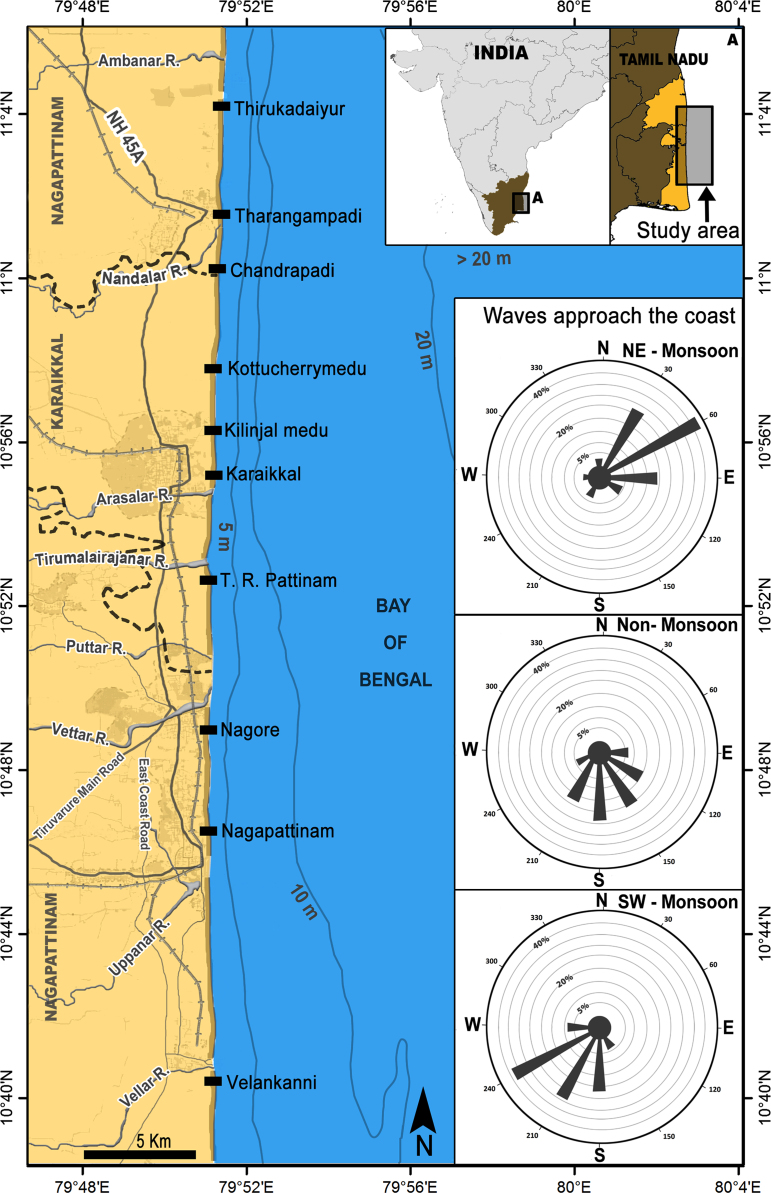
Figure illustrates location map of the study site. The wind rose diagram exhibit waves approach the coast in northeast (NE), southwest (SW) and non-monsoon conditions.

This report includes morphometric, hydrodynamic and granulometric dataset of the sandy beaches along the central Tamil Nadu coast, India. The study area extents upto 51 km of the coastal stretch, located between Thirukadaiyur in the north and Velankanni in the south. It lies in Karaikkal district and also parts of Nagapattinam district. Totally, ten stations were fixed with an approximate interval of 5 km, between Thirukadaiyur and Velankanni (Fig. 1). The beach and wave characteristic of these ten sites is presented in Table 1. Data was collected in this region on a monthly basis from January 2011 to December 2011 which includes monsoonal seasons such as northeast (NE) monsoon (November to February), non-monsoon (March to June) and southwest (SW) monsoon (July to October). The entire dataset is displayed in ten Excel files. Each file represents each station of the present study area. The Excel file contains nearshore data such as wave period (T), breaker wave height (Hb), longshore current velocity (Vc), surf zone width (W) and particle settling velocity (Vs). Subsequently, the dataset contains types of breaker waves and beach morphodynamic state. We provided field photographs that support these results with respect to the seasonal change. Alongside, the dataset includes mean, sorting, skewness and kurtosis value of tidal, berm and backshore sediments which is derived from both graphical methods and method of moments. Moreover, it includes monthly data of heavy and light minerals present in the tide, berm and backshore region.

**Table 1:** Beach and wave characteristics of the present study area.

S.No.	Station	Average beach width (m)	Predominant wind direction	Mean wind speed (Km/h)	Mean tide level (m) (High tide / Low tide)	Mean foreshore slope (deg)
1	Thirukadaiyur	83	NE (45°), SW (225°), SE (135°)	20	0.63 / 0.25	4.25
2	Tharangampadi	32	NE (45°), SW (225°), SE (135°)	17	0.59 / 0.21	4.19
3	Chandrapadi	61	NE (45°), SW (225°), SE (135°)	18	0.60 / 0.22	4.36
4	Kottucherrymedu	63	NE (45°), SW (225°), SE (135°)	18	0.62 / 0.23	3.92
5	Kilinjal medu	58	NE (45°), SW (225°), SE (135°)	19	0.58 / 0.22	4.35
6	Karaikkal	181	NE (45°), SW (225°), SE (135°)	20	0.61 / 0.23	4.56
7	T.R. Pattinam	87	NE (45°), SW (225°), SE (135°)	17	0.64 / 0.26	4.54
8	Nagore	88	NE (45°), SW (225°), SE (135°)	19	0.65 / 0.25	4.51
9	Nagapattinam	96	NE (45°), SW (225°), SE (135°)	21	0.69 / 0.27	4.20
10	Velankanni	72	NE (45°), SW (225°), SE (135°)	19	0.67 / 0.24	4.52

### 1.2 Data acquisition methods

The nearshore wave parameters are estimated on a monthly basis from January 2011 to December 2011 at spring low-tide period. Breaking wave height (Hb) is measured by the calibrated leveling with millimeter (mm) scale accuracy. The height of the waves at breaking point is computed from the line of sight to the wave crest and horizon by fixing the leveling staff at low tide [1]. Significant wave height is obtained at the one third of the successive breaking waves. Alongside, wave period (T) is measured by the time taken for passing two crests at a particular point. From that, predominant wave period is estimated from the average time period of 300 successive waves [2], [3]. The buoyant plate floating a distance in 2 min beyond the breaker point is employed to record the longshore current velocity (Vc) [4]. Similarly, total station is employed to evaluate the surf zone width (W) by calculating distance between the boat at the breaker zone and the low tide region. The data on particle settling velocity (Vs), breaker wave type and beach morphodynamic state are estimated from OceaN WavE Tool (ONWET) statistical software [5].

Samples of surficial beach sediments were collected at the tide, berm and backshore region with the help of an Aluminum grabber with a single open edge. On the whole, about 360 samples were packed and properly labeled for laboratory analysis. After soaking it in water, the samples were perturbed by a mechanical stirrer to disaggregate them and to withdraw the clay fractions. Further, the organic and inorganic contents were removed by the addition of 30% Hydrogen Peroxide (H_2_O_2_) and 10% of Hydrochloric acid (HCl) [6]. Coning and quartering method was employed to extract 100*g* of pre-treated sands from the each sample. A Ro-top sieve shaker was used to sieve the treated samples with quarter phi interval mesh grids ranging from +40 to +230 ASTM units. The graphical method [7] and method of moments [8] are used to extricate the textural behavior of these sieve fractions. Further, heavy and light minerals were isolated by the addition of bromoform (specific gravity is 2.89 and molecular weight 252.73 g/mol) as per the standard procedure [9]. The isolated heavy and light minerals were weighted and tabulated.

## 2 Processed data in supplementary files

Seasonal variation of beach and nearshore dynamics is displayed in field photograph in a TIFF format. Nearshore data of each station is provided in a table format. Classification of Breaker wave type and beach morphodynamic state is represented by table. Particle size distribution is represented by the statistical measures of mean, sorting, skewness and kurtosis in a table format. Similarly, the weight percentage of light and heavy minerals is presented in table format.
